# Combination therapy with Nifuroxazide and Lenvatinib exerts superior anti-hepatocellular carcinoma effect by enhancing the anti-tumor immune response

**DOI:** 10.3389/fonc.2026.1937787

**Published:** 2026-08-28

**Authors:** Jing Yang, Jiaxin Geng, Yue Yin, Kangle Wang, Leyao Tian, Jingyi Wang, Huijie Jia, Lei Wang, Yongxi Zhang, Tiesuo Zhao

**Affiliations:** 1 Department of Medical Technology, Shangqiu Medical College, Shangqiu, China; 2 Xinxiang Engineering Technology Research Center of Immune Checkpoint Drug for Liver-Intestinal Tumors, Henan Medical University, Xinxiang, China; 3 Xinxiang Key Laboratory of Tumor Vaccine and Immunotherapy, Henan Medical University, Xinxiang, China; 4 Department of Urology, Xinxiang Central Hospital, Xinxiang, China; 5 Xinxiang Key Laboratory for Tumor Radiotherapy and Targeted Therapy, the Third Affiliated Hospital of Henan Medical University, Xinxiang, China

**Keywords:** anti-tumor immune response, combination therapy, hepatocellular carcinoma, Lenvatinib, Nifuroxazide, tumor-bearing mice

## Abstract

**Background:**

Lenvatinib is a first-line multi-target tyrosine kinase inhibitor for hepatocellular carcinoma (HCC) that exerts anti-angiogenic effects by blocking VEGF signaling. However, its clinical efficacy is often compromised by adaptive resistance. Nifuroxazide, an anti-diarrheal agent, has been identified by our group as a potent inhibitor of PD-L1 expression in HCC.

**Objective:**

This study aimed to evaluate the therapeutic potential and underlying molecular mechanisms of nifuroxazide combined with lenvatinib against HCC.

**Methods:**

*In vitro*, HCC cell proliferation, colony formation, and migration were assessed, and Western blot was used to detect p-STAT3, STAT3, PD-L1, and VEGF levels. *In vivo*, antitumor efficacy was evaluated in mouse xenograft models. Tumor tissues were analyzed by immunohistochemistry, TUNEL, and H&E staining, while flow cytometry was used to measure CD4^+^ and CD8^+^ T-cell proportions in the tumor microenvironment, peripheral blood, and spleen.

**Results:**

The combination synergistically inhibited HCC cell proliferation and migration, and downregulated PD-L1, p-STAT3, and VEGF expression. It also markedly suppressed tumor growth, induced apoptosis, and enhanced CD4^+^ and CD8^+^ T-cell infiltration and distribution.

**Conclusions:**

Nifuroxazide potentiates lenvatinib-induced antitumor activity by regulating the STAT3/PD-L1 axis and enhancing antitumor immune responses. This combination provides a promising therapeutic strategy for HCC.

## Introduction

Liver cancer remains one of the leading causes of cancer-related mortality, with mortality rates showing an upward trend in many countries. Hepatocellular carcinoma (HCC) accounts for approximately 90% of all primary liver cancer cases and represents a major global health challenge (1). HCC is characterized as a highly vascularized tumor (2). Tumor vascularization is achieved primarily through two mechanisms: sprouting angiogenesis and the co-option of existing vessels by expanding tumor tissue. Among these, sprouting angiogenesis is the principal pathway for neovascularization in solid tumors, significantly facilitating tumor growth and metastasis. Angiogenesis, defined as the formation of new blood vessels from pre-existing endothelial cells (ECs), is essential for supplying tumors with nutrients and oxygen while removing metabolic waste (3). Histologically, HCC tissue comprises tumor cells, a basement membrane, and the surrounding stroma. The rapid proliferation of HCC cells induces a hypoxic microenvironment within the tumor, which strongly stimulates angiogenesis (4, 5).

Vascular endothelial growth factor (VEGF) plays a pivotal role in vasculogenesis and angiogenesis and encompasses a family of related genes, including VEGF-B, VEGF-C, and placental growth factor (PlGF). Under physiological conditions, VEGF signaling is a key regulator of vasculogenesis, angiogenesis, and lymphangiogenesis, and is critical for vascular and lymphatic system development, tissue repair, and homeostasis maintenance (6–8). In cancer patients, however, VEGF overexpression is a key driver of the molecular pathogenesis underlying tumor growth and metastasis (9). Consequently, targeting the VEGF pathway has emerged as a crucial therapeutic strategy in oncology. VEGF-targeted therapies, represented by monoclonal antibodies and tyrosine kinase inhibitors (TKIs), have demonstrated significant potential across various tumor types, offering new hope for patients (10). Lenvatinib, a multi-target TKI, inhibits the activity of several kinases critical for carcinogenesis, including vascular endothelial growth factor receptors (VEGFRs), thereby blocking multiple signaling pathways essential for tumor growth and angiogenesis. The core mechanism of Lenvatinib’s antitumor action lies in inhibiting tumor angiogenesis and reducing the formation of neovessels that supply nutrients to the tumor, thereby limiting its expansion (11). Given that HCC relies not only on vasculature for nutrients but also exploits blood vessels to disseminate thousands of cancer cells to initiate metastasis (2), the anti-angiogenic mechanism of Lenvatinib is vital for inhibiting HCC progression. Studies indicate that Lenvatinib can induce a state of hypoxia and nutrient deprivation within tumor cells, ultimately leading to cell death (12). Thus, Lenvatinib has become a highly promising therapeutic option for HCC (13).

However, the development and progression of HCC are influenced by multifactorial mechanisms. To achieve optimal clinical benefits, researchers are actively exploring novel combination strategies for HCC (14–16). Programmed death-ligand 1 (PD-L1) is a critical immune checkpoint molecule that binds to programmed death-1 (PD-1) on T cells, inhibiting T cell activation and leading to T cell exhaustion; this interaction is essential for maintaining immune homeostasis (17). In tumor patients, PD-L1 is frequently overexpressed within the tumor microenvironment (TME), and its interaction with PD-1 on T cells has long been recognized as a major mechanism of tumor immune escape (18). Immune checkpoint inhibitors that block the PD-1/PD-L1 interaction can effectively activate T lymphocytes and elicit durable antitumor responses (19), achieving tremendous success in clinical oncology. Emerging evidence reveals that combinations of anti-PD-1/PD-L1 agents with anti-VEGF drugs demonstrate safety and efficacy in advanced HCC, representing a viable and promising therapeutic strategy (20). Consequently, the search for novel PD-1/PD-L1 inhibitors continues. However, *de novo* drug development is a lengthy process, and despite substantial financial investment, outcomes are often uncertain (21, 22). Therefore, “drug repurposing”—identifying new therapeutic uses for existing drugs—has garnered increasing attention (23). Nifuroxazide, an antibiotic traditionally used to treat infectious diarrhea, was previously confirmed by our group to not only effectively inhibit the proliferation and migration of HCC cells but also suppress PD-L1 expression, thereby exerting anti-HCC effects (24). In this study, using both *in vitro* and *in vivo* tumor-bearing models, we further demonstrate that the combination of Nifuroxazide and Lenvatinib exerts superior anti-HCC efficacy by effectively inhibiting the expression of VEGF and PD-L1.

## Materials and methods

### Cell lines and reagents

The H22 and HepG2 cell lines were obtained from the Xinxiang Key Laboratory of Tumor Vaccine and Immunotherapy. Nifuroxazide was purchased from Aladdin (N129550-5g). Lenvatinib was obtained as Lenvatinib Mesylate Capsules from Eisai (China) Co., Ltd.

### Experimental animals

Specific pathogen-free (SPF) female C57BL/6 mice, aged 6–8 weeks, were purchased from Henan Specks Biotechnology Co., Ltd. The mice were housed under pathogen-free conditions with a 12 h light/dark cycle and provided with free access to food and water.

### Cell counting kit-8 assay

A CCK-8 kit (MCE, MedChemExpress, Monmouth Junction, NJ, USA) was used to assess cell viability. HepG2 cells were seeded into 96-well plates at a density of 1 × 10^4^ cells/well and incubated for 16 h. The cells were then treated with Lenvatinib (10 µg/mL), Nifuroxazide (5 µg/mL), or the combination of Lenvatinib (10 µg/mL) and Nifuroxazide (5 µg/mL) for 24 h. The culture medium was discarded, and the cells were gently washed with PBS. According to the manufacturer’s instructions, 10 µL of CCK-8 reagent was added to each well, and the cells were incubated for an additional 2 h. The absorbance (OD value) at 450 nm was measured using a microplate reader (Thermo Fisher Scientific).

### Colony formation assay

HepG2 cells were seeded into 6-well plates at a density of 2 × 10³ cells/well and incubated for 16 h. Subsequently, the cells were treated with Lenvatinib (10 µg/mL), Nifuroxazide (5 µg/mL), or the combination (Lenvatinib 10 µg/mL + Nifuroxazide 5 µg/mL). After 24 h of culture, the medium was replaced with fresh medium containing the corresponding drugs. The cells were cultured until visible cell colonies formed with appropriate size. The medium was then discarded, and the cells were washed with PBS. One milliliter of cell fixation solution was added to each well and incubated for 30 min. After removing the fixation solution, crystal violet staining solution was added to each well and incubated for another 30 min. The cells were then washed, dried, and photographed.

### Wound healing assay

HepG2 cells were seeded into 6-well plates at a density of 3.5 × 10^5^ cells/well and cultured for 16 h. A straight scratch (“-” shape) was made in the center of each well using a sterile pipette tip. Subsequently, the cells were treated with Lenvatinib (5 µg/mL), Nifuroxazide (2.5 µg/mL), or the combination (Lenvatinib 5 µg/mL + Nifuroxazide 2.5 µg/mL) and cultured for an additional 24 or 48 h. Cell migration was observed under a microscope (Nikon, Japan).

### Western blot analysis

Proteins were extracted from cells or tissues using RIPA lysis buffer (Beyotime Institute of Biotechnology, Shanghai, China). 4× protein loading buffer (Beijing Solarbio Science & Technology) was added, and the samples were heated in a 95 °C metal bath for 13 min to prepare SDS protein samples. The protein samples were separated by 10% SDS-PAGE gel electrophoresis and transferred to PVDF membranes (EMD Millipore, USA). The membranes were then incubated with TBST solution containing 5% skimmed milk and 0.05% Tween-20 for 2 h. Subsequently, the membranes were incubated with primary antibodies diluted according to the manufacturer’s instructions (p-STAT3 1:1000, CST; CyclinD1 1:2000, SANTA; STAT3 1:1000, BioLegend; MMP2 1:2000, Proteintech; Tubulin 1:50000, Proteintech; PD-L1 1:5000, Proteintech; VEGF 1:12000, Proteintech) overnight at 4 °C. The following day, after thorough washing, the membranes were incubated with the corresponding secondary antibodies (ZSGB-BIO, 1:5000) at room temperature for 1 h, followed by another wash. Finally, enhanced chemiluminescence (ECL) reagent (Beyotime, China) was added to the membranes, and specific protein bands were detected using a chemiluminescence imager (Vilber Fusion, France). ImageJ software was used for semi-quantitative analysis of the images.

### Establishment and treatment of the tumor-bearing mouse model

A hepatocellular carcinoma tumor-bearing mouse model was established by subcutaneously inoculating each mouse with 1 × 10^6^ H22 cells. Seven days later, the mice were randomly divided into four groups: PBS group, Lenvatinib group (200 µg/mouse), Nifuroxazide group (200 µg/mouse), and the combination group (Lenvatinib 200 µg/mouse + Nifuroxazide 200 µg/mouse). Treatments were administered according to the group assignments: Nifuroxazide was administered via intratumoral injection, and Lenvatinib was administered via intragastric gavage. All treatments were administered once daily for a total of seven times. The third day after the last treatment, the mice were randomly sacrificed, and tumor tissues, spleens, and peripheral blood were collected for the detection of relevant indicators.

### Immunofluorescence

Paraffin sections of tumor tissues were placed in citrate buffer at 95 °C for 15 min for antigen retrieval. Subsequently, 3% hydrogen peroxide was added to the sections and incubated for 10 min. The sections were washed thoroughly with PBS and incubated with blocking solution containing 5% goat serum at room temperature for 15 min. Primary antibodies (CD3 1:100, OmnimAbs; CD4 1:200, CST; CD8 1:800, CST; CD86 1:200, Affinity; PD-L1 1:500, Proteintech; Ki67 1:1000, Proteintech; PCNA 1:500, SANTA) were added to the sections, which were then incubated overnight at 4 °C in the dark. The next day, the sections were brought to room temperature for 2 h, followed by the addition of HRP-conjugated secondary antibodies (1:1000, Abways) and incubation at room temperature in the dark for 30 min. After washing, DAPI staining solution (Beyotime) was added and incubated for 10 min. The sections were washed again, mounted with anti-fluorescence quenching mounting medium (Beyotime), and observed under a laser confocal microscope (AR1+, Nikon).

### TUNEL assay

Paraffin sections of tumor tissues were treated with Proteinase K (DNase-free) and incubated at 37 °C for 60 min. The sections were then washed with PBS, dried, and incubated with TUNEL detection solution (Beyotime Institute of Biotechnology) at 37 °C in the dark for 60 min according to the manufacturer’s instructions. Finally, the sections were washed, incubated with DAPI staining solution (Beyotime) at room temperature for 10 min, mounted with anti-fluorescence quenching mounting medium (Beyotime), and observed under a confocal microscope (AR1+, Nikon).

### Immunohistochemistry

Tumor tissue sections were placed in boiling citrate solution (Solarbio) for 15 min. Goat serum (Boster Biological Technology) was then added to the sections and incubated for 30 min. After washing, primary antibodies (Ki67 1:4000, Proteintech; PCNA 1:500, SANTA) were added and incubated overnight at 4 °C. The next day, the sections were brought to room temperature for 30 min, washed, and incubated with reaction enhancement solution at 37 °C for 20 min. The sections were washed again and incubated with enzyme-labeled goat anti-rabbit IgG polymer (ZSGB-BIO) at 37 °C for 20 min. After thorough washing, DAB chromogenic solution was added to each section. The reaction was terminated when brown particles were observed under the microscope. The sections were then incubated with hematoxylin (Solarbio) for 20 s, rinsed for bluing, dehydrated with graded alcohols, cleared with xylene, and mounted with neutral gum. Staining results were observed under an optical microscope.

### Hematoxylin and eosin staining

Tumor tissue sections were immersed in hematoxylin solution (Solarbio) and incubated at room temperature for 5 min, followed by rinsing with running water to remove excess dye. Subsequently, the sections were incubated with 1% eosin solution (Solarbio, Beijing, China) at room temperature for 2 min, followed by another rinse with running water to remove unbound dye. Finally, the sections were dehydrated through a graded ethanol series, cleared with xylene, mounted with neutral gum (Solarbio, Beijing, China), and observed under an optical microscope.

### Flow cytometry

Spleen cell suspensions or peripheral blood cell suspensions from mice were prepared. Red blood cells were lysed using red blood cell lysis buffer (Shanghai Buyontai Biotechnology Research Institute, China), and the cells were counted. Antibodies against CD3, CD4, and CD8 (all from BioLegend) were added to 1 × 10^6^ cells according to the antibody instructions and incubated at 4 °C in the dark for 30 min. The cells were then washed with PBS buffer. Finally, the cells were resuspended in 300 µL of PBS buffer, and the cell proportions were detected using a flow cytometer (CytoFLEX, Beckman).

### Statistical analysis

Statistical data are expressed as the mean ± SD of three independent experiments. Statistical analysis was performed using GraphPad Prism 8.0.2. One-way analysis of variance (ANOVA) and Student’s t-test were used for comparisons. A *P*-value < 0.05 was considered statistically significant.

## Results

### Nifuroxazide combined with Lenvatinib significantly inhibits the proliferation and migration of hepatocellular carcinoma cells

**Figure 1:**
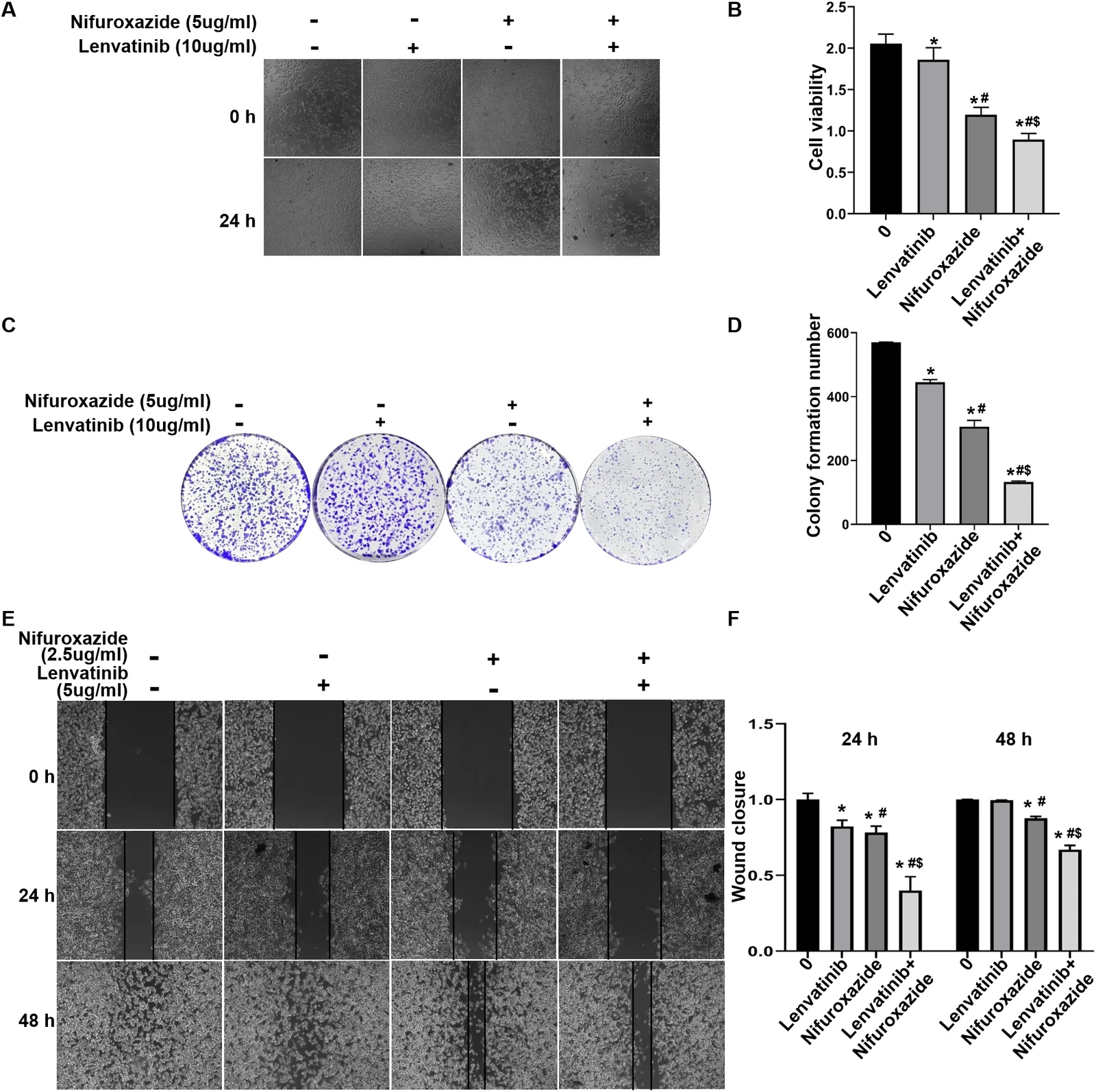
Nifuroxazide combined with Lenvatinib inhibits the proliferation and migration of hepatocellular carcinoma cells. HepG2 cells were treated with 5 µg/mL Nifuroxazide and 10 µg/mL Lenvatinib to evaluate cell proliferation using CCK-8 and colony formation assays. Additionally, HepG2 cells were treated with 5 µg/mL Nifuroxazide and 10 µg/mL Lenvatinib to evaluate cell migration using a wound healing assay. **(A, B)** Cell morphology and CCK-8 statistical analysis after 24 h of treatment with Nifuroxazide and Lenvatinib. **(C, D)** Representative images of colony formation and statistical analysis showing the effect of Nifuroxazide combined with Lenvatinib on cell proliferation. **(E, F)** Representative images of the wound healing assay and statistical analysis showing the effect on cell migration after 24 h or 48 h of treatment with Nifuroxazide and Lenvatinib. Data are expressed as mean ± SD (n=3). *P < 0.05 vs. the control group; ^#^*P* < 0.05 vs. the Lenvatinib group; ^$^*P* < 0.05 vs. the Nifuroxazide group.

First, we explored the effects of Nifuroxazide combined with Lenvatinib on the proliferation and migration of HCC cells. Results from CCK-8 and colony formation assays showed that, compared to the untreated group, treatment with either Nifuroxazide or Lenvatinib alone inhibited cell proliferation, with the combination treatment exhibiting the most potent inhibitory effect on proliferation (**Figures 1A–D**). Wound healing assay results indicated that, compared to the control group, Nifuroxazide treatment alone effectively inhibited tumor cell migration at both 24 and 48 hours. While Lenvatinib treatment alone showed an inhibitory effect on migration at 24 hours, it did not significantly affect migration at 48 hours. Importantly, compared to the single-agent groups, the combination of Nifuroxazide and Lenvatinib significantly inhibited tumor cell migration at both 24 and 48 hours (**Figures 1E, F**).

### Effects of Nifuroxazide combined with Lenvatinib on the expression of tumor-related proteins in HCC cells

**Figure 2:**
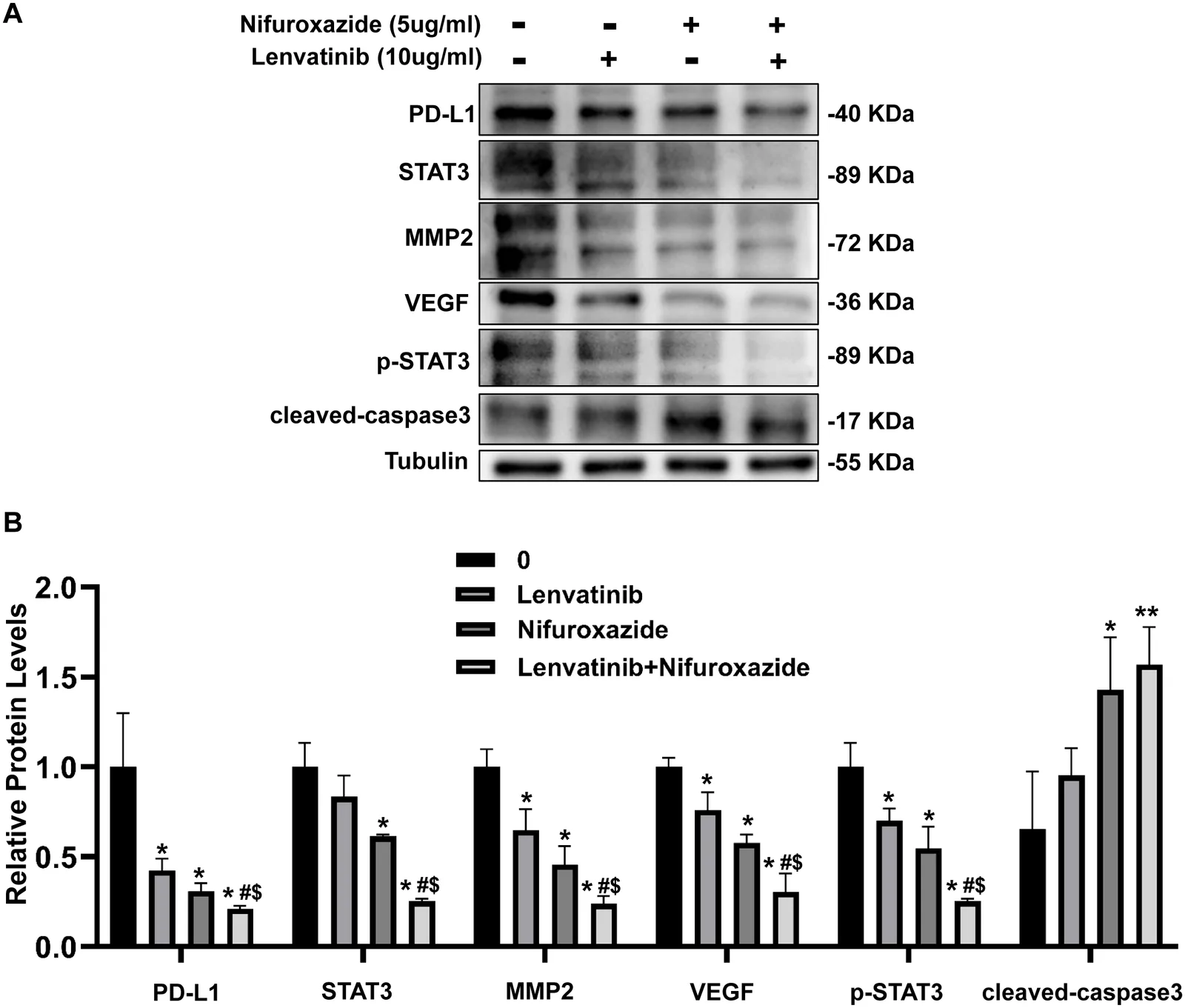
Effect of Nifuroxazide combined with Lenvatinib on the expression levels of related proteins in hepatocellular carcinoma cells. HepG2 cells were treated with 5 µg/mL Nifuroxazide and 10 µg/mL Lenvatinib for 24 h. Western blot was used to detect the expression of PD-L1, STAT3, MMP2, VEGF, p-STAT3, and cleaved caspase-3, followed by quantitative statistical analysis. Data are expressed as mean ± SD (n=3). *P < 0.05 vs. the control group; ^#^*P* < 0.05 vs. the Lenvatinib group; ^$^*P* < 0.05 vs. the Nifuroxazide group.

Subsequently, we examined the effects of Nifuroxazide combined with Lenvatinib on the expression of tumor-related proteins, such as PD-L1 and VEGF, in HCC cells using Western blot. The results showed that, compared to the untreated group, both Nifuroxazide and Lenvatinib monotherapies effectively inhibited the expression of PD-L1, p-STAT3, VEGF, and MMP2 in HCC cells. Nifuroxazide treatment alone effectively increased the expression of the apoptosis-related protein cleaved caspase-3, whereas Lenvatinib treatment alone did not significantly affect cleaved caspase-3 levels. Furthermore, compared to the monotherapy groups, the combination treatment further reduced the expression levels of the tumor-related proteins PD-L1, p-STAT3, VEGF, and MMP2. However, the expression level of cleaved caspase-3 in the combination group showed no significant difference compared to the Nifuroxazide monotherapy group (**Figures 2A, B**).

### Nifuroxazide combined with Lenvatinib significantly inhibits tumor growth in HCC-bearing mice

**Figure 3:**
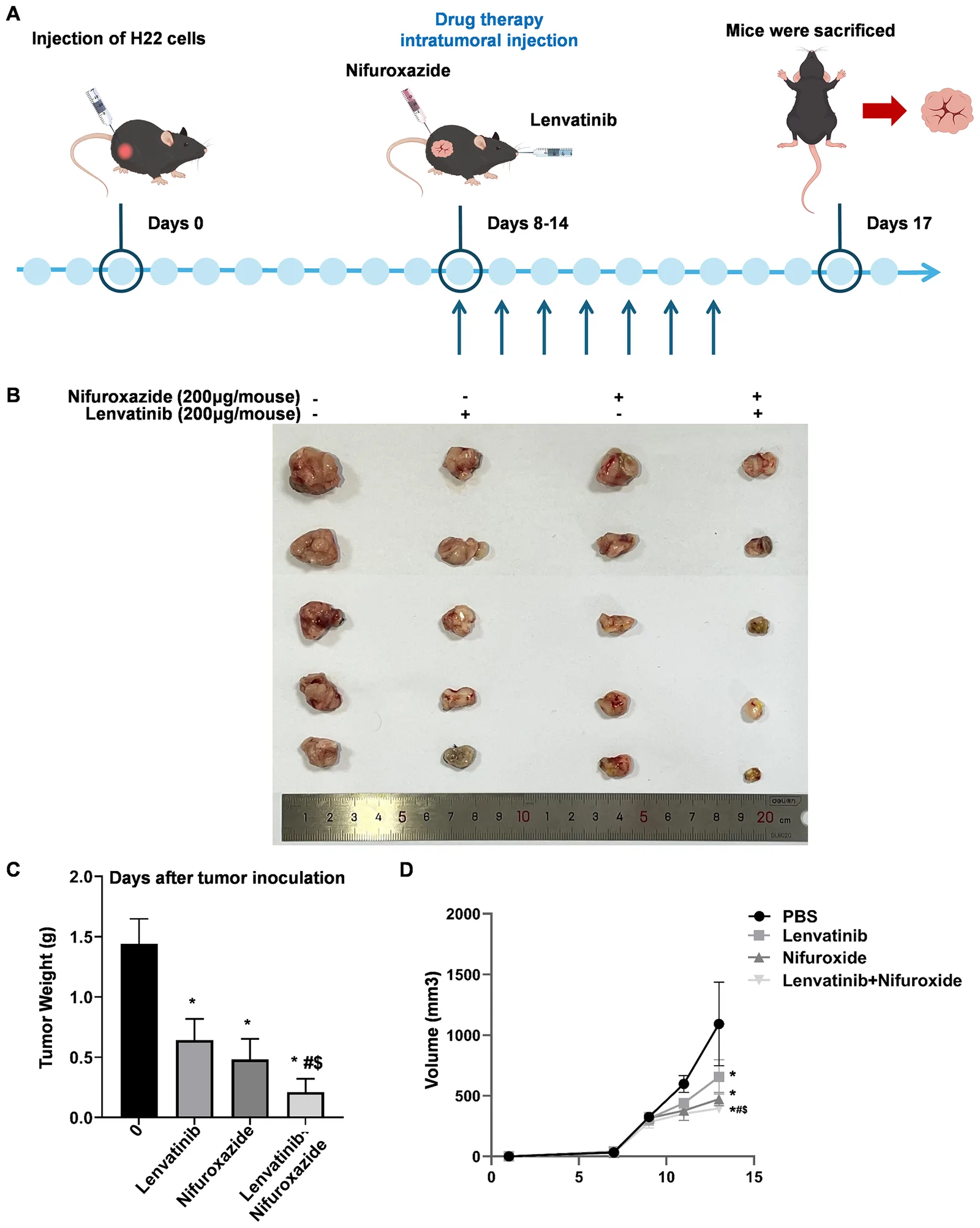
Effect of Nifuroxazide combined with Lenvatinib treatment on tumor growth in hepatocellular carcinoma-bearing mice. A hepatocellular carcinoma tumor-bearing mouse model was established. After successful modeling, the mice were randomly divided into PBS, Lenvatinib, Nifuroxazide, and Nifuroxazide combined with Lenvatinib groups. Treatments were administered as follows: the PBS group received intratumoral injection of 100 µL PBS per mouse; the Lenvatinib group received 200 µg Lenvatinib per mouse via intragastric gavage; the Nifuroxazide group received 200 µg Nifuroxazide per mouse via intratumoral injection; the combination treatment group received 200 µg Lenvatinib (intragastric gavage) and 200 µg Nifuroxazide (intratumoral injection). Treatments were administered once daily for 7 consecutive days, and tumor volume was recorded during the treatment period. Mice were sacrificed 3 days after the last treatment for analysis of relevant indicators. **(A)** Schematic diagram of the experimental workflow. **(B, C)** Photographs of tumors from tumor-bearing mice and statistical analysis of tumor weights. **(D)** Tumor growth curve of the tumor-bearing mice. Data are expressed as mean ± SD (n=5). *P < 0.05 vs. the control group; ^#^*P* < 0.05 vs. the Lenvatinib group; ^$^*P* < 0.05 vs. the Nifuroxazide group.

We further evaluated the antitumor effects of Nifuroxazide combined with Lenvatinib using a tumor-bearing animal model (**Figure 3A**). The results indicated that, compared to the control group, treatment with either Nifuroxazide or Lenvatinib alone delayed the increase in tumor volume in the mice. Tumor weight results also showed that mice in the Nifuroxazide or Lenvatinib monotherapy groups had significantly lighter tumors than the control group. Compared to any other group, the tumors in the combination treatment group were the lightest (**Figures 3B, C**). The tumor volume growth rate was the slowest in the combination treatment group compared to the monotherapy groups (**Figure 3D**).

### Nifuroxazide combined with Lenvatinib significantly inhibits the expression of proliferation-related proteins and promotes apoptosis in tumor tissues

**Figure 4:**
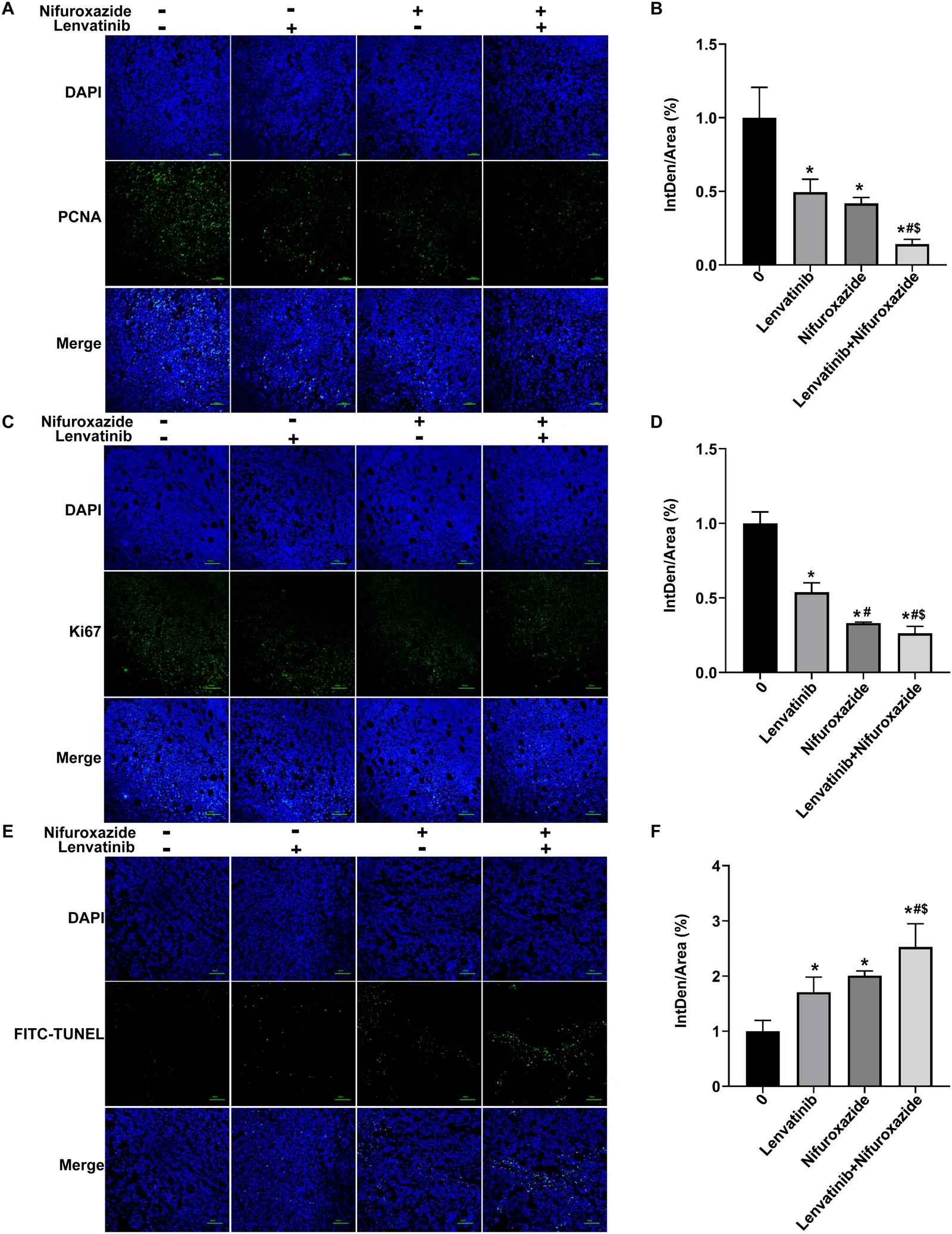
Effect of Nifuroxazide combined with Lenvatinib treatment on cell proliferation and apoptosis in tumor tissues of tumor-bearing mice. **(A, B)** Immunofluorescence detection of the expression of the proliferation-related protein PCNA in tumor tissues and statistical analysis. **(C, D)** Immunofluorescence detection of the expression of the proliferation-related protein Ki67 in tumor tissues and statistical analysis. **(E, F)** TUNEL assay detection of apoptosis in tumor tissues and statistical analysis. Data are expressed as mean ± SD (n=3). *P < 0.05 vs. the control group; ^#^*P* < 0.05 vs. the Lenvatinib group; ^$^*P* < 0.05 vs. the Nifuroxazide group.

We detected the effects of Nifuroxazide combined with Lenvatinib on the expression of the proliferation-related proteins Ki67 and PCNA, as well as tumor cell apoptosis, using immunofluorescence and TUNEL assays, respectively. The results showed that, compared to the control group, treatment with either Nifuroxazide or Lenvatinib alone effectively inhibited the expression of Ki67 and PCNA in tumor tissues, with the combination treatment demonstrating the strongest inhibitory effect (**Figures 4A–D**). Similarly, TUNEL assay results revealed that, compared to any other group, Nifuroxazide combined with Lenvatinib treatment significantly increased tumor cell apoptosis (**Figures 4E, F**).

### Effects of Nifuroxazide combined with Lenvatinib on the expression of tumor-related proteins in HCC tissues

**Figure 5:**
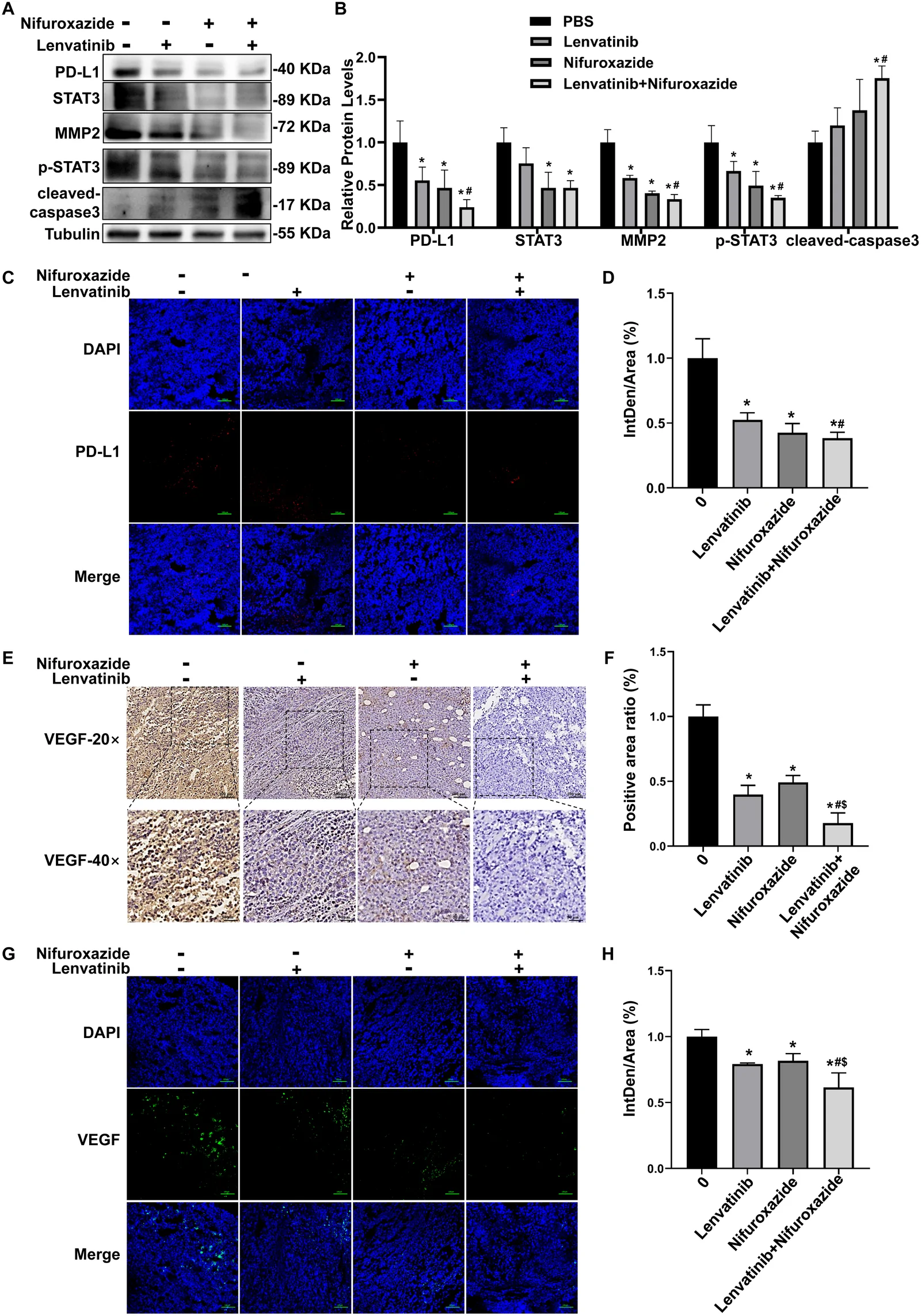
Effect of Nifuroxazide combined with Lenvatinib treatment on the expression of related proteins in tumor tissues. **(A, B)** Western blot detection and statistical analysis of the effects of Nifuroxazide combined with Lenvatinib on the expression of PD-L1, STAT3, MMP2, VEGF, p-STAT3, and cleaved caspase-3 in tumor tissues. **(C, D)** Immunofluorescence detection and statistical analysis of PD-L1 expression in tumor tissues. **(E, F)** Immunohistochemistry detection and statistical analysis of VEGF expression in tumor tissues. **(G, H)** Immunofluorescence detection and statistical analysis of VEGF expression in tumor tissues. Data are expressed as mean ± SD (n=3). *P < 0.05 vs. the control group; ^#^*P* < 0.05 vs. the Lenvatinib group; ^$^*P* < 0.05 vs. the Nifuroxazide group.

Next, we investigated the effects of Nifuroxazide combined with Lenvatinib on the expression of tumor-related proteins, such as PD-L1, in HCC tissues via Western blot. Western blot results indicated that, compared to the PBS group, both Nifuroxazide and Lenvatinib monotherapies effectively inhibited the expression of PD-L1, p-STAT3, VEGF, and MMP2, but did not significantly affect cleaved caspase-3 expression. However, we observed that, compared to the PBS group and the Lenvatinib monotherapy group, the combination treatment further inhibited the expression of PD-L1, MMP2, and p-STAT3, although no significant difference was observed compared to the Nifuroxazide monotherapy group. Compared to any other group, the Nifuroxazide and Lenvatinib combination treatment significantly increased the expression level of the apoptosis-related protein cleaved caspase-3 (**Figures 5A, B**). Subsequently, we further detected PD-L1 expression in tumor tissues via immunofluorescence, and the results were consistent with the trends shown in the Western blot (**Figures 5C, D**). Immunohistochemistry and immunofluorescence detection of VEGF in tumor tissues revealed that both Nifuroxazide and Lenvatinib monotherapies effectively inhibited VEGF expression, with the combination treatment showing the most obvious inhibitory effect (**Figures 5E–H**).

### Nifuroxazide combined with Lenvatinib significantly increases the ratio of T lymphocytes in the peripheral blood and spleen of tumor-bearing mice

**Figure 6:**
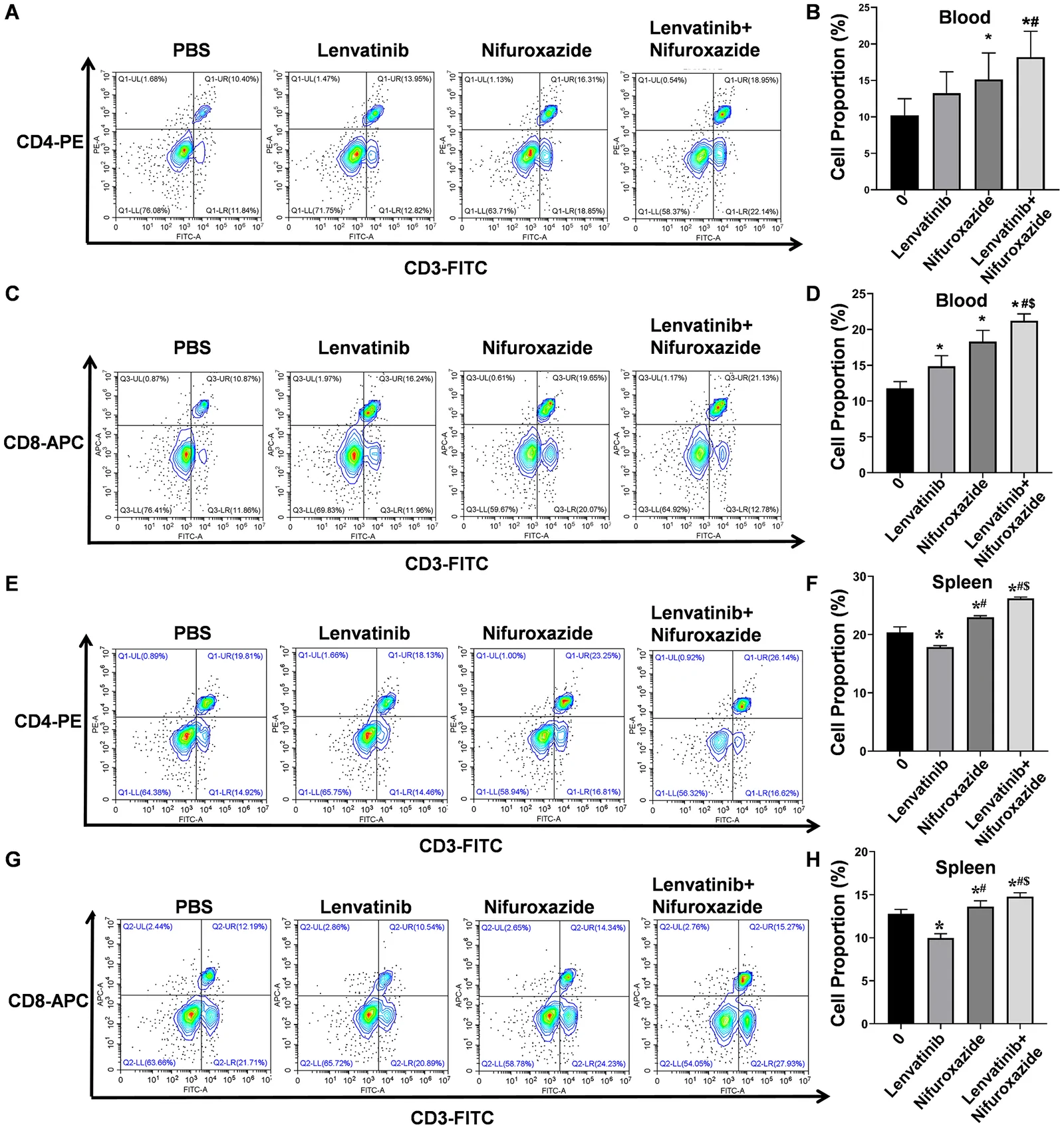
Effect of Nifuroxazide combined with Lenvatinib treatment on the proportions of T lymphocyte subsets in the peripheral blood and spleen of tumor-bearing mice. Flow cytometry was used to detect the ratios of T lymphocyte subsets in the peripheral blood and spleen of tumor-bearing mice. **(A, B)** The ratio of CD4^+^ T lymphocytes in the peripheral blood of tumor-bearing mice and statistical analysis. **(C, D)** The ratio of CD8^+^ T lymphocytes in the peripheral blood of tumor-bearing mice and statistical analysis. **(E, F)** The ratio of CD4^+^ T lymphocytes in the spleen of tumor-bearing mice and statistical analysis. **(G, H)** The ratio of CD8^+^ T lymphocytes in the spleen of tumor-bearing mice and statistical analysis. Data are expressed as mean ± SD (n=3). *P < 0.05 vs. the control group; ^#^*P* < 0.05 vs. the Lenvatinib group; ^$^*P* < 0.05 vs. the Nifuroxazide group.

To assess the impact of Nifuroxazide combined with Lenvatinib on the antitumor immune response in tumor-bearing mice, we measured the ratio of T lymphocytes in the peripheral blood and spleen using flow cytometry. The results showed that Nifuroxazide treatment effectively increased the ratio of CD4^+^ T lymphocytes in the peripheral blood, whereas Lenvatinib treatment did not cause significant changes. Compared to the Lenvatinib monotherapy group, the ratio of CD4^+^ T lymphocytes in the peripheral blood was significantly increased in the combination treatment group (**Figures 6A, B**). Additionally, compared to the PBS group, both Nifuroxazide and Lenvatinib treatments showed an effect in increasing the ratio of CD8^+^ T lymphocytes, with the combination treatment group exhibiting the highest ratio of CD8^+^ T lymphocytes in the peripheral blood (**Figures 6C, D**). The spleen, as a vital peripheral immune organ and a site of immune response, was also analyzed. We found that the ratios of both CD4^+^ and CD8^+^ T lymphocytes were significantly elevated after treatment with either Nifuroxazide or Lenvatinib. Compared to any other group, the combination treatment group showed the highest ratios of CD4^+^ and CD8^+^ T lymphocytes in the spleen (**Figures 6E–H**). This suggests that Nifuroxazide combined with Lenvatinib treatment exerts an effect in enhancing the antitumor immune response in tumor-bearing mice.

### Nifuroxazide combined with Lenvatinib effectively improves immune cell infiltration in tumor tissues

**Figure 7:**
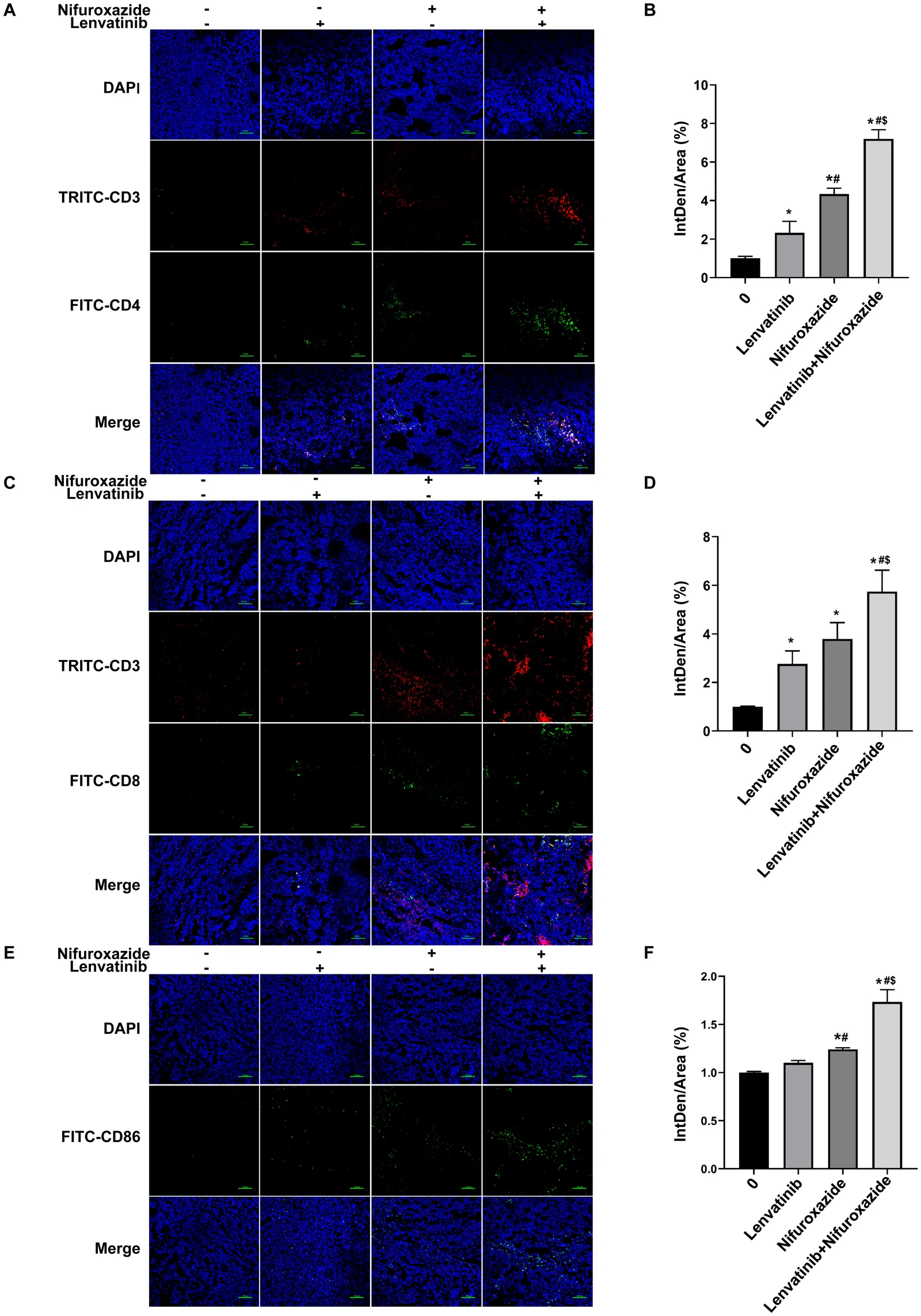
Effect of Nifuroxazide combined with Lenvatinib treatment on immune cell infiltration in the tumor tissues of tumor-bearing mice. **(A, B)** Immunofluorescence detection and statistical analysis of the infiltration of CD4^+^ T lymphocytes in tumor tissues. **(C, D)** Immunofluorescence detection and statistical analysis of the infiltration of CD8^+^ T lymphocytes in tumor tissues. **(E, F)** Immunofluorescence detection and statistical analysis of the infiltration of CD86^+^ cells in tumor tissues. Data are expressed as mean ± SD (n=3). *P < 0.05 vs. the control group; ^#^*P* < 0.05 vs. the Lenvatinib group; ^$^*P* < 0.05 vs. the Nifuroxazide group.

Immune cell infiltration within tumor tissues is highly correlated with patient prognosis; therefore, we explored the effects of Nifuroxazide combined with Lenvatinib on immune cell infiltration in the tumor tissues of tumor-bearing mice. As shown in the **Figure 7**, compared to the PBS group, the tumor tissues of mice in the Nifuroxazide or Lenvatinib monotherapy groups exhibited infiltration by a greater number of CD4^+^ and CD8^+^ T lymphocytes. The combination treatment resulted in the highest number of infiltrating T lymphocytes within the tumor tissues (**Figures 7A–D**). CD86 is one of the characteristic surface markers of M1 macrophages. Finally, we detected the expression of CD86 in tumor tissues following Nifuroxazide and Lenvatinib treatment. We found that, compared to the PBS group, Nifuroxazide treatment increased the ratio of CD86^+^ cells in tumor tissues, whereas Lenvatinib treatment did not have a significant effect. Among all groups, the combination treatment group exhibited the highest ratio of CD86^+^ cells in tumor tissues (**Figures 7E, F**).

## Discussion

Anti-angiogenic therapy constitutes a cornerstone of targeted treatment for HCC. However, single-agent therapy in this field faces significant bottlenecks, with drug resistance being a particularly prominent issue. Consequently, combination therapy has emerged as a primary direction for breakthrough in recent years (25–27). In this study, we demonstrated that Nifuroxazide, a clinical antibiotic, combined with Lenvatinib more effectively inhibited the expression of VEGF and PD-L1 in tumor tissues of HCC-bearing mice. This combination suppressed tumor growth and enhanced the host’s antitumor immune response.

Angiogenesis plays a pivotal role in tumor progression. The regulation of angiogenesis within tumor tissue is a multi-dimensional process governed by the interaction between tumor cells and various stromal cells, mediated by a multitude of bioactive products, such as cytokines and growth factors (28). Studies indicate that dysregulation of the VEGF signaling pathway can trigger various pathological conditions, including tumor angiogenesis, as well as cardiovascular and ocular diseases. Increased VEGF expression promotes tumor growth, invasion, and metastasis (29), making it a prominent therapeutic target in multiple malignancies, including HCC. Lenvatinib, an oral small-molecule tyrosine kinase inhibitor (TKI), exerts its antitumor effects primarily by blocking pathways including VEGF. Beyond its potent anti-angiogenic properties, Lenvatinib also inhibits tumor cell proliferation and induces apoptosis (30). Consistent with these findings, our study confirmed that Lenvatinib effectively inhibits the proliferation and migration of HCC cells. However, despite the significant advantages Lenvatinib has shown in treating advanced HCC, its clinical efficacy remains suboptimal (31). Research has confirmed that over 60% of HCC patients develop resistance to Lenvatinib within one year of treatment, with only a small subset achieving long-term clinical benefits (32). Therefore, enhancing the antitumor efficacy of Lenvatinib and identifying effective combination strategies represent urgent and critical issues in the field.

In recent years, driven by breakthroughs in the medical field, immunotherapy—particularly the application of immune checkpoint inhibitors—has become a focal point of clinical research and exploration. PD-L1 expressed on tumor cells inhibits the function of cytotoxic T cells and promotes their apoptosis, leading to tumor immune escape (33). Studies have demonstrated that blocking PD-L1 can, to a certain extent, improve the anti-HCC efficacy of Lenvatinib (34). Current combination regimens for unresectable hepatocellular carcinoma include atezolizumab plus bevacizumab, durvalumab plus tremelimumab, and lenvatinib combined with PD-1 inhibitors. These clinically approved regimens exert anti-tumor effects through systemic administration via anti-angiogenic and immune blockade mechanisms (35–37). Although these strategies have been validated to prolong patient survival, they still have several limitations, including high medical costs, bleeding risks, and acquired drug resistance caused by persistent activation of the STAT3 pathway (38–40). Our group previously confirmed that Nifuroxazide, a repurposed clinical drug, effectively inhibits STAT3 and PD-L1 expression in tumor cells and exerts a synergistic anti-HCC effect when combined with radiotherapy (24). However, its potential to enhance the efficacy of Lenvatinib against HCC remained unclear. Our study reveals that at the cellular level, the combination of Nifuroxazide and Lenvatinib results in more significant inhibition of HCC cell proliferation and migration compared to monotherapies. Similarly, in tumor-bearing models, the combination therapy more effectively suppressed HCC tumor growth. This mechanism is likely associated with the superior ability of the combination treatment to inhibit the expression of STAT3, PD-L1 and VEGF. Furthermore, it is well established that the infiltration of CD4^+^ and CD8^+^ T lymphocytes into the tumor microenvironment is closely correlated with patient prognosis and therapeutic efficacy (41–43). Our results showed that compared to the monotherapy groups, the tumor tissues of mice in the combination treatment group exhibited significantly higher infiltration of CD4^+^ and CD8^+^ T lymphocytes. This suggests that the combination of Nifuroxazide and Lenvatinib synergistically enhances the antitumor immune response in HCC-bearing mice.

In summary, this study demonstrates that Nifuroxazide combined with Lenvatinib exerts a more significant inhibitory effect on HCC. This combination suppresses the expression of PD-L1 and VEGF within tumor tissues and enhances the antitumor immune response in tumor-bearing mice, providing a solid experimental basis for the development of combination strategies for HCC in clinical practice.

## Limitations of the Present Study

Admittedly, several limitations remain in the current work, which warrant further in-depth investigation in our subsequent research. First, although this study verified that the combination of nifuroxazide and lenvatinib could markedly downregulate the protein levels of p-STAT3 and PD-L1, rescue experiments including STAT3 knockdown and overexpression were not performed. In follow-up studies, we will establish stable cell lines with STAT3 knockdown and overexpression, as well as PD-L1 knockdown cell models, to further clarify whether the anti-tumor efficacy of nifuroxazide combined with lenvatinib is dependent on the p-STAT3/PD-L1 signaling pathway. Second, only the HepG2 hepatocellular carcinoma (HCC) cell line was used for *in vitro* cellular verification, and repeated experiments in other HCC cell lines were lacking, which constitutes a notable limitation. For *in vivo* anti-tumor evaluation, we merely adopted the subcutaneous H22 hepatoma xenograft mouse model. Despite its easy operation and wide application, this model exhibits prominent drawbacks in clinical translational value. As mouse-derived hepatoma cells, H22 cells differ greatly from human HCC cells in biological properties and tumor microenvironment. This model fails to recapitulate patient-specific tumor heterogeneity, immune landscapes and authentic clinical disease progression, restricting the extrapolation of our *in vivo* findings to clinical practice. Therefore, multiple HCC cell lines and diverse animal models will be incorporated into our future research to facilitate the clinical translation of our results. Third, nifuroxazide suffers from poor oral absorption and low bioavailability; it only exerts weak local efficacy in the intestinal tract and cannot achieve the effective concentration required for STAT3 inhibition in HCC lesions via systemic administration (44, 45). Accordingly, intratumoral injection was applied in this study to achieve high *in situ* drug concentration within tumors and characterize its modulatory effects on the tumor immune microenvironment. Lenvatinib is an orally administered anti-tumor drug approved for clinical use (31). Hence, a differentiated therapeutic regimen combining intratumoral nifuroxazide injection with oral lenvatinib was designed herein. Nevertheless, the optimal administration strategy and dosage for this combination require further optimization. Distinct delivery routes lead to completely separated *in vivo* drug exposure profiles for the two agents. In particular, intratumoral injection of nifuroxazide deviates from its feasible clinical administration mode. To promote clinical translation, our group will systematically explore alternative combined delivery regimens of the two drugs, and conduct structural modification of nifuroxazide to improve its oral absorption efficiency. Fourth, our previous studies have confirmed that 5 µg/mL nifuroxazide exerts potent anti-HCC activity and inhibits STAT3 expression (24), while 10 µg/mL lenvatinib can induce HCC cell apoptosis, suppress cell proliferation and reduce VEGF protein expression (46). For this reason, the combination of 5 µg/mL nifuroxazide and 10 µg/mL lenvatinib was selected for anti-HCC cellular experiments in the present study. Further investigations are still needed to determine the optimal effective and safe dosage of this combination, optimize administration routes, and improve the oral bioavailability of nifuroxazide before its clinical application.

## Data Availability

The original contributions presented in the study are included in the article/**Supplementary Material**. Further inquiries can be directed to the corresponding authors.
